# Supplementary material for: Desmin and Plectin Recruitment to the Nucleus and Nuclei Orientation Are Lost in Emery-Dreifuss Muscular Dystrophy Myoblasts Subjected to Mechanical Stimulation
Source: Cells. 2024 Jan 16;13(2):162. doi: 10.3390/cells13020162 (PMC10814836; doi:10.3390/cells13020162)
Supplement: Supplementary file 1 [file cells-13-00162-s001.zip › Supplementary Materials 08.01.24.pdf]

Article

Desmin and plectin recruitment to the nucleus and nuclei orientation are lost in Emery-Dreifuss Muscular Dystrophy myoblasts subjected to mechanical stimulation

Vittoria Cenni [1,2], Camilla Evangelisti [3], Spartaco Santi [1,2], Patrizia Sabatelli [1,2], Simona Neri [4], Marco Cavallo[5], Giovanna Lattanzi [1,2,*], Elisabetta Mattioli [1,2,*].

[1] CNR Institute of Molecular Genetics "Luigi Luca Cavalli-Sforza", Unit of Bologna, Bologna, Italy.

vittoria.cenni@cnr.it (V.C.); spartaco.santi@cnr.it (S.S.); patrizia.sabatelli@area.bo.cnr.it (P.S.); giovanna.lattanzi@cnr.it (G.L.); e.mattioli@area.bo.cnr.it (E.M.).

[2] IRCCS Istituto Ortopedico Rizzoli, Bologna, Italy.

[3] Cellular signalling Laboratory, Department of Biochemical and Neuromotor Sciences, Alma Mater Studiorum, University of Bologna, Bologna, Italy. camilla.evangelisti@unibo.it (C.E.).

[4] Medicine and Rheumatology Unit, IRCCS Istituto Ortopedico Rizzoli, Bologna, Italy. simona.neri@ior.it (S.N.).

[5] Shoulder-elbow surgery Unit, IRCCS Istituto Ortopedico Rizzoli, Bologna, Italy. marco.cavallo@ior.it (M.C.).

* Correspondence : e.mattioli@area.bo.cnr.it; giovanna.lattanzi@cnr.it.

Supplementary Materials

Figure S1

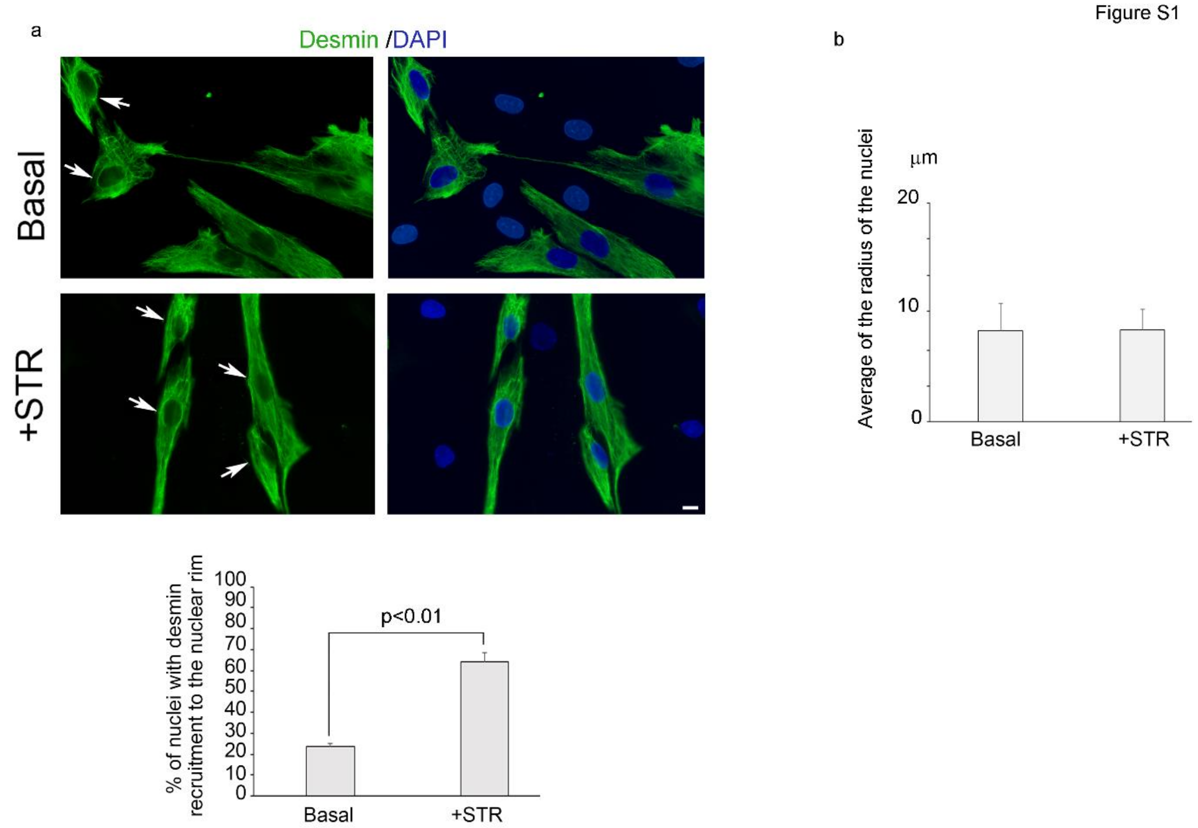

Figure S1. Desmin localization in human myoblasts under basal condition (Basal) or after cyclic stretching (+STR). a) Immunofluorescence analysis of desmin (green). Nuclei with desmin localization at the nuclear envelope were indicate with arrows (white arrows). Statistical analysis of % of nuclei with desmin recruitment to the rim was reported in the graph below. b) Average of the radius of the nuclei under basal condition (basal) and after mechanical stretching (+STR) was reported in the graph. DAPI (blue) was used to counterstain cell nuclei. Three biological replicates were used in each experiment and statistically significant differences ($p < 0.01$) between values are indicated. Scale bars, 10 μm.

Figure S2

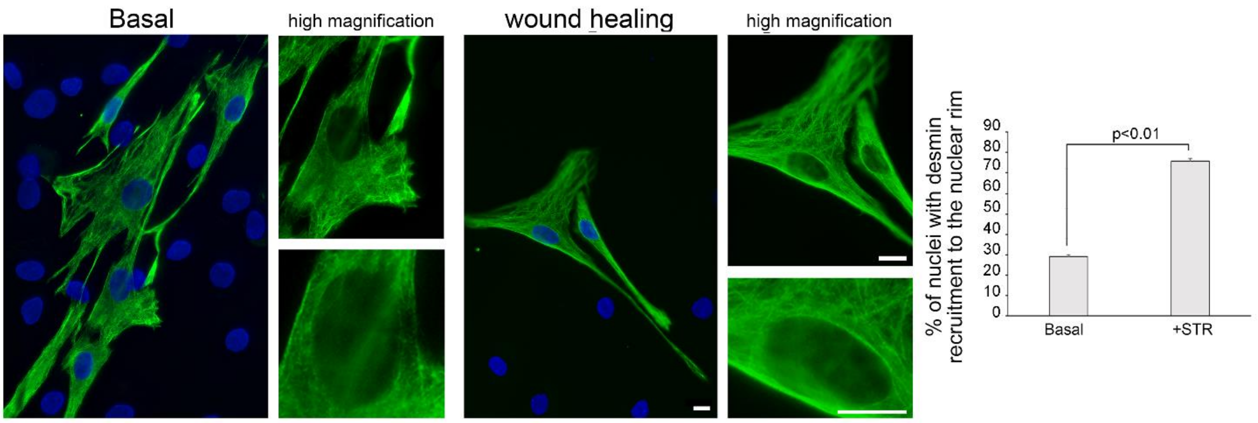

Figure S2. Desmin is recruited to the nuclear rim during myoblast migration. Immunofluorescence analysis of desmin (green) was performed in basal conditions (Basal) and after wound healing assay (wound healing). High magnification images of desmin (green) on the right. DAPI (blue) was used to counterstain cell nuclei. Percentage of nuclei with desmin recruitment to the rim was reported in the graph on the right. Scale bars, 10 μm. Three biological replicates were used in each experiment and statistically significant differences ($p < 0.01$) between values are indicated.

Figure S3

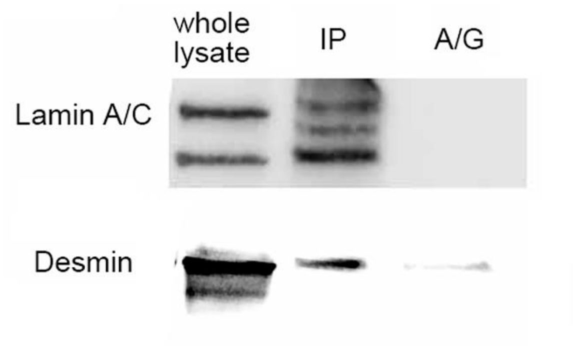

Figure S3. Co-immunoprecipitation of desmin and lamin A/C in myoblasts after mechanical stretching. Total lysate (whole lysate) of stretched myoblasts, immunoprecipitated proteins (IP) of 500 μg of protein and negative control with nonspecific immunoglobulins form Santa Cruz (A/G). Immunoprecipitates were blotted against anti-lamin A/C antibody (E1 Santa Cruz) over-night and immunoblotting with anti-lamin A/C (E1 Santa Cruz) and anti-desmin (Abcam Ab15200) were performed.

Figure S4

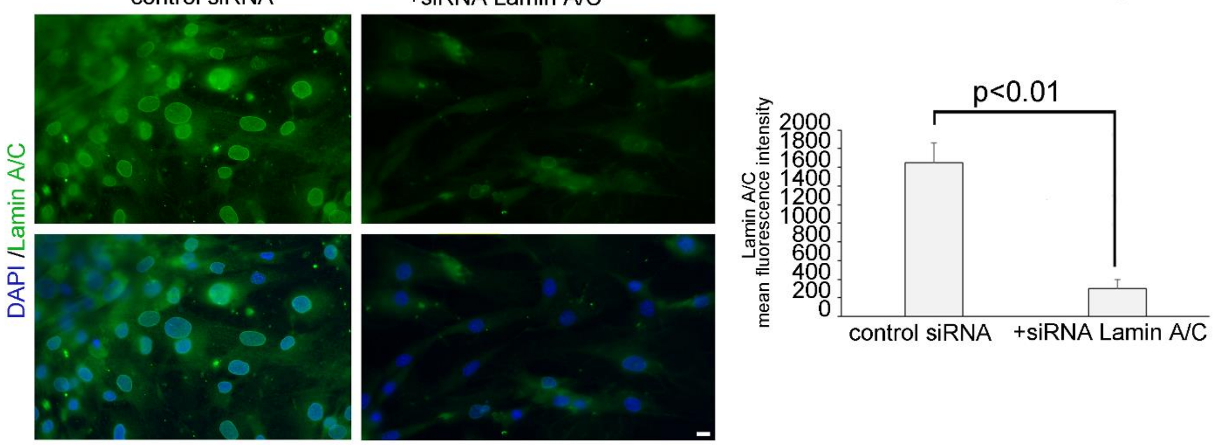

Figure S4. Lamin A/C reduction in control myoblasts subjected to siRNA Lamin A/C. Immunofluorescence analysis of lamin A/C (green) in scrambled control and after silencing of lamin A/C

(siRNA Lamin A/C) of human myoblasts subjected to mechanical stretching. Statistical analysis of lamin A/C mean fluorescence intensity, was reported in the graph on the right. DAPI (blue) was used to counterstain cell nuclei. Scale bars, 10 µm. Three biological replicates were used in each experiment and statistically significant differences ($p < 0.01$) between values are indicated.

Figure S5

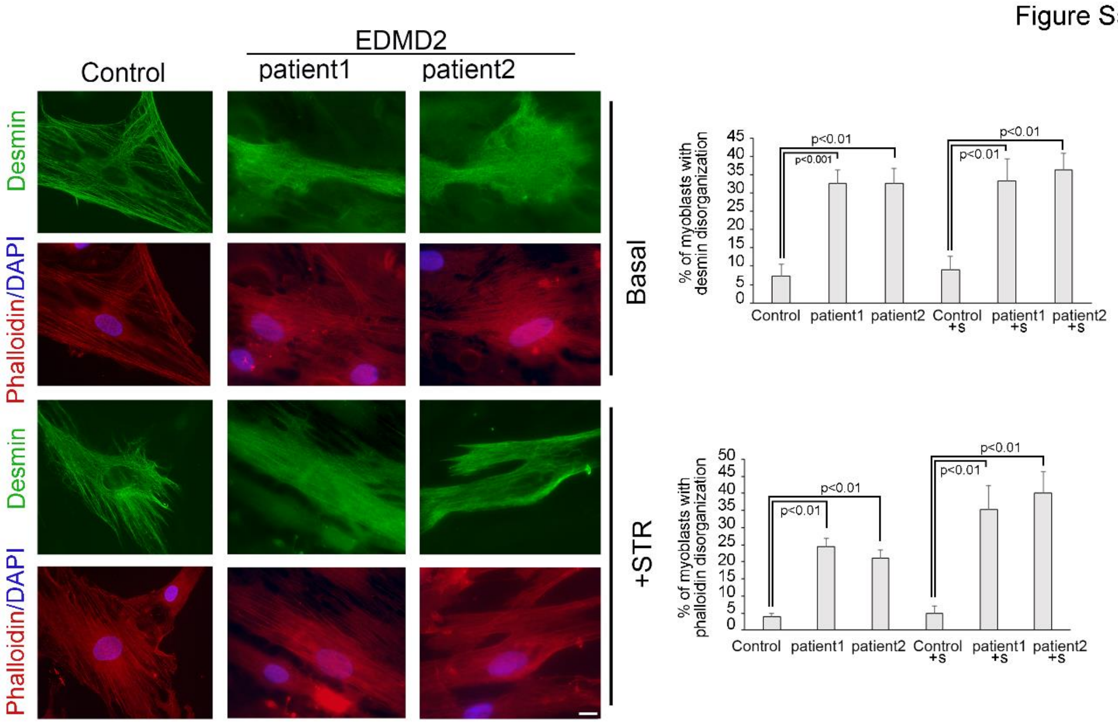

Figure S5. Defects of desmin and phalloidin organization were observed in EDMD2 myoblasts. Immunofluorescence analysis of desmin (green) and phalloidin (red) in human myoblasts from healthy donors and EDMD2 patients, under basal condition (Basal) or after mechanical stretching (+STR). Percentage of myoblasts with desmin and phallodin filaments disorganization in the cytoplasmic region, were reported in the graphs on the right. DAPI (blue) was used to counterstain cell nuclei. Scale bars, 10 µm. Three biological replicates were used in each experiment and statistically significant differences ($p < 0.01$) between values are indicated.

Figure S6

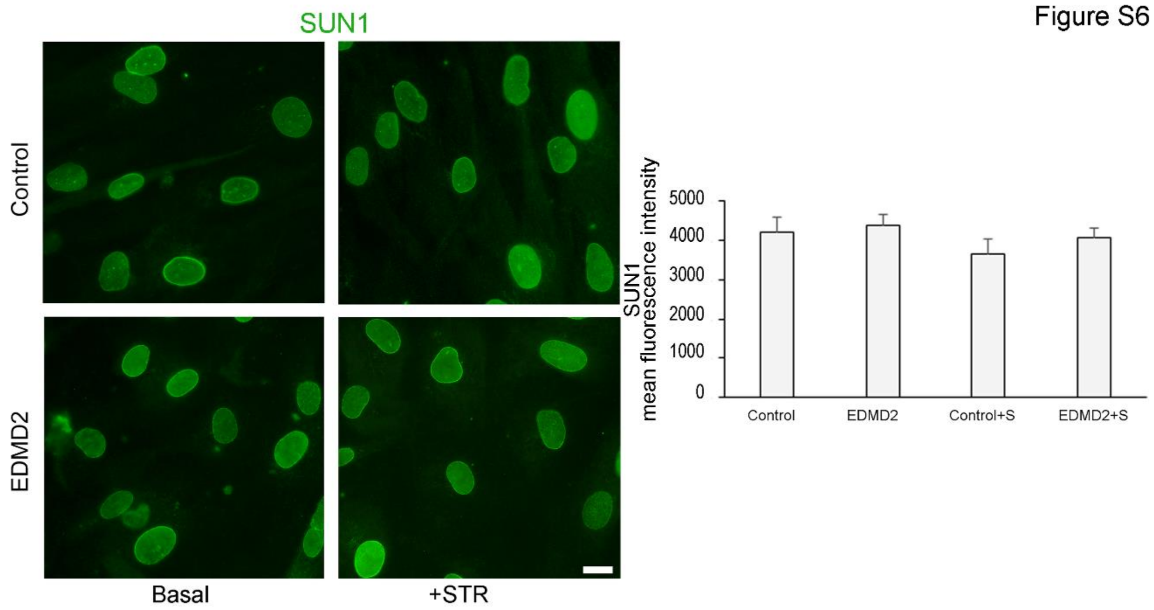

Figure S6. No difference of SUN1 expression was observed in EDMD2 myoblasts with respect to control myoblasts. Immunofluorescence analysis of SUN1 (green) in human myoblasts from healthy donor and EDMD2 patient, in basal condition (Basal) or after mechanical stretching (+STR). SUN1 mean fluorescence intensity was reported in the graph on the right. Scale bars, 10 μm. Three biological replicates were used in each experiment.

a

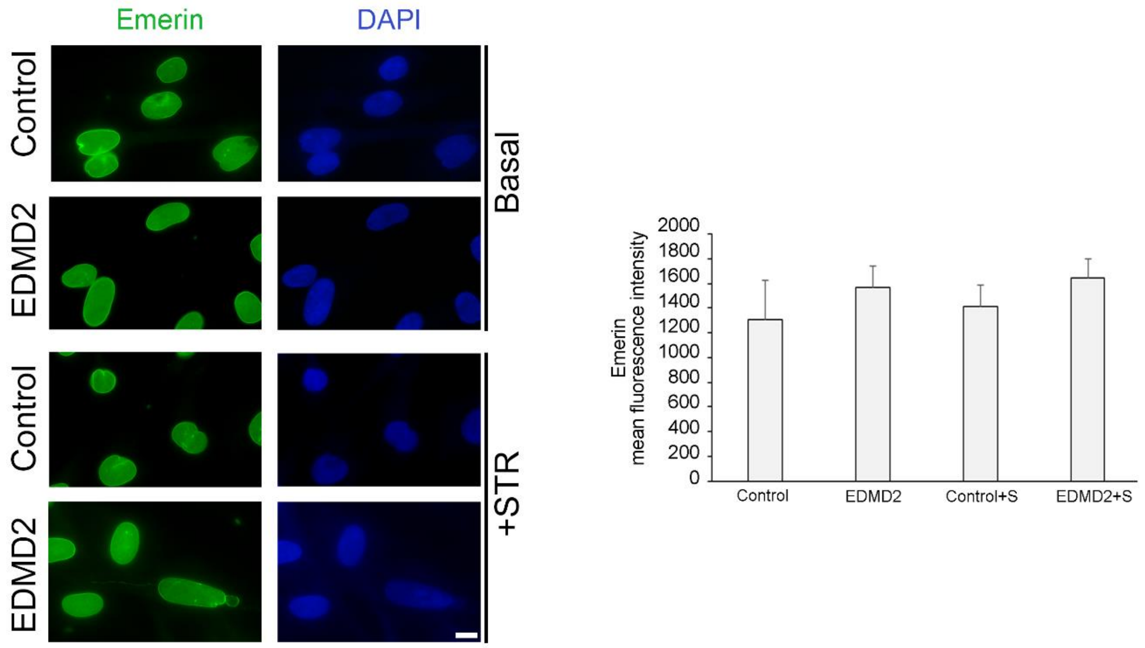

b

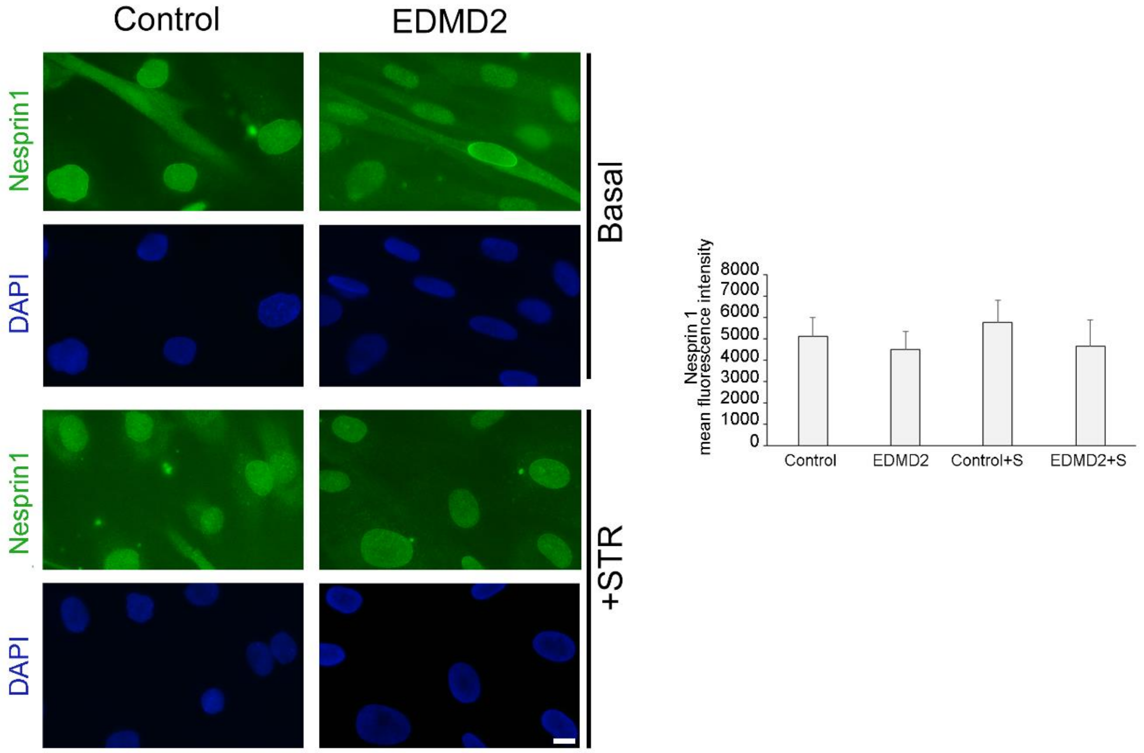

Figure S7. Immunolocalization of emerin and nesprin 1 in healthy donors myoblasts is similar to EDMD2 myoblasts, both in basal condition (Basal) or after mechanical stretching (+STR). a) Immunofluorescence analysis of emerin (green). Mean of fluorescence intensity was reported in the graph on the right. b) Immunofluorescence analysis of nesprin 1 (green). Nesprin 1 mean fluorescence intensity

was reported in the graphs on the right. DAPI (blue) was used to counterstain cell nuclei. Scale bars, 10 µm. Three biological replicates were used in each experiment.

Figure S8

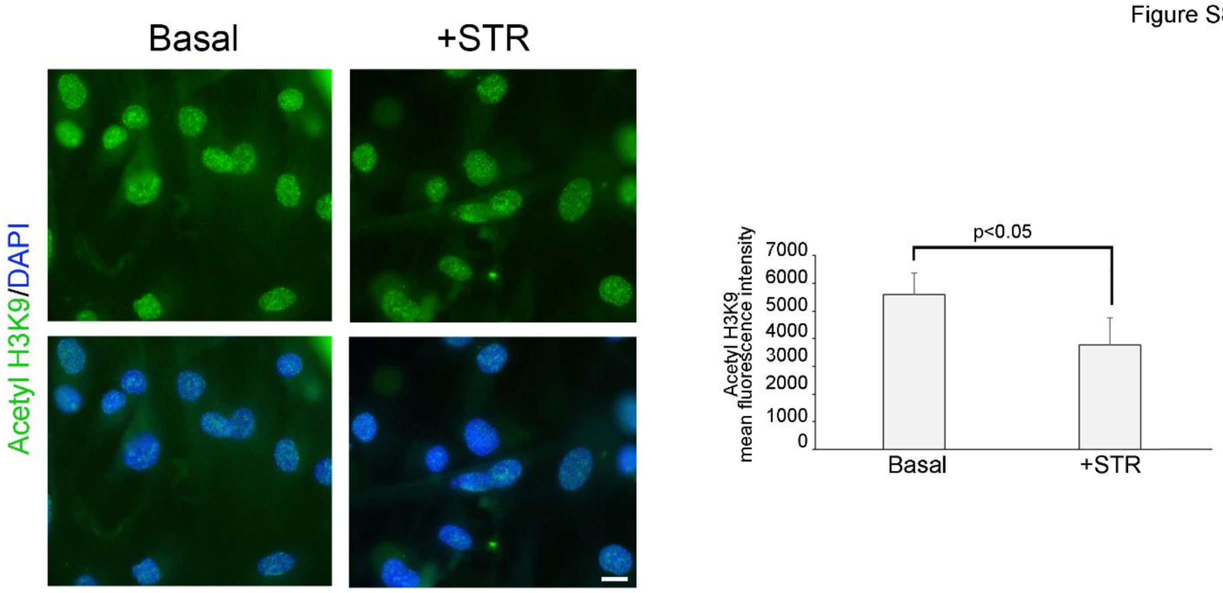

Figure S8. AcetylH3K9 expression was reduced in healthy donors subjected to mechanical stretching. Immunofluorescence analysis of acetyl H3K9 (green) in human myoblasts from healthy donors under basal condition (Basal) or after cyclic stretching (+STR). Acetyl H3K9 fluorescence intensity was reported in the graph on the right. Scale bars, 10 µm. Three biological replicates were used in each experiment and statistically significant differences ($p < 0.05$) between values are indicated.
