# Supplementary figures and images for: Desmin and Plectin Recruitment to the Nucleus and Nuclei Orientation Are Lost in Emery-Dreifuss Muscular Dystrophy Myoblasts Subjected to Mechanical Stimulation
Source: Cells. 2024 Jan 16;13(2):162. doi: 10.3390/cells13020162 (PMC10814836; doi:10.3390/cells13020162)

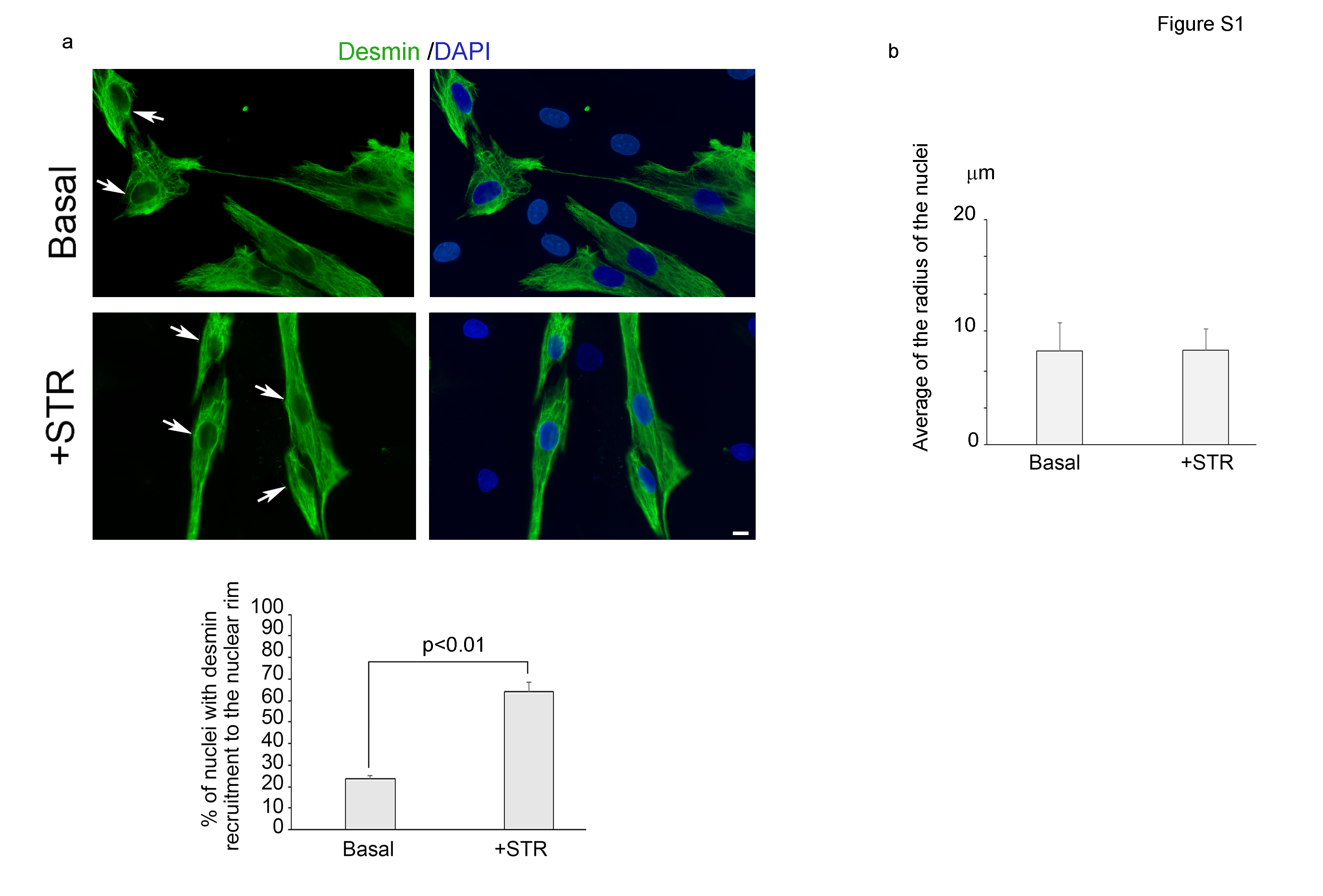

Supplement: Supplementary file 1 [file cells-13-00162-s001.zip › figure s1.tif]

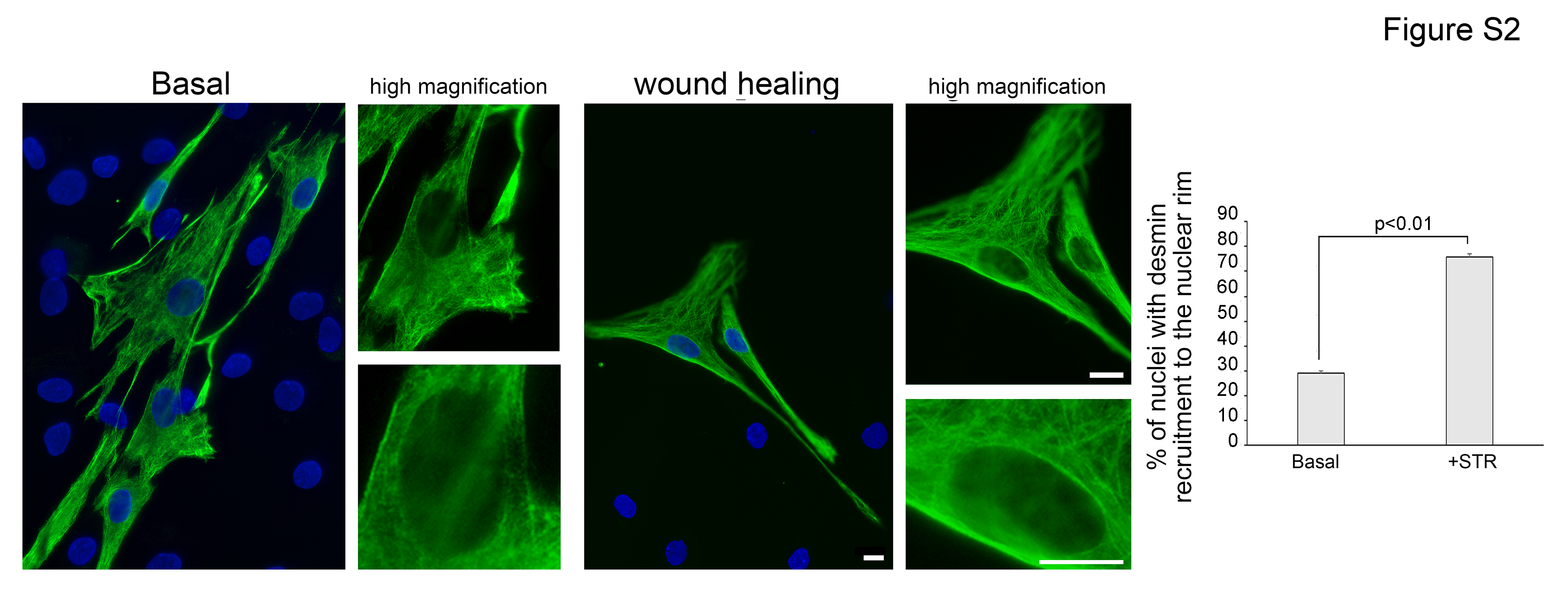

Supplement: Supplementary file 1 [file cells-13-00162-s001.zip › figure S2.tif]

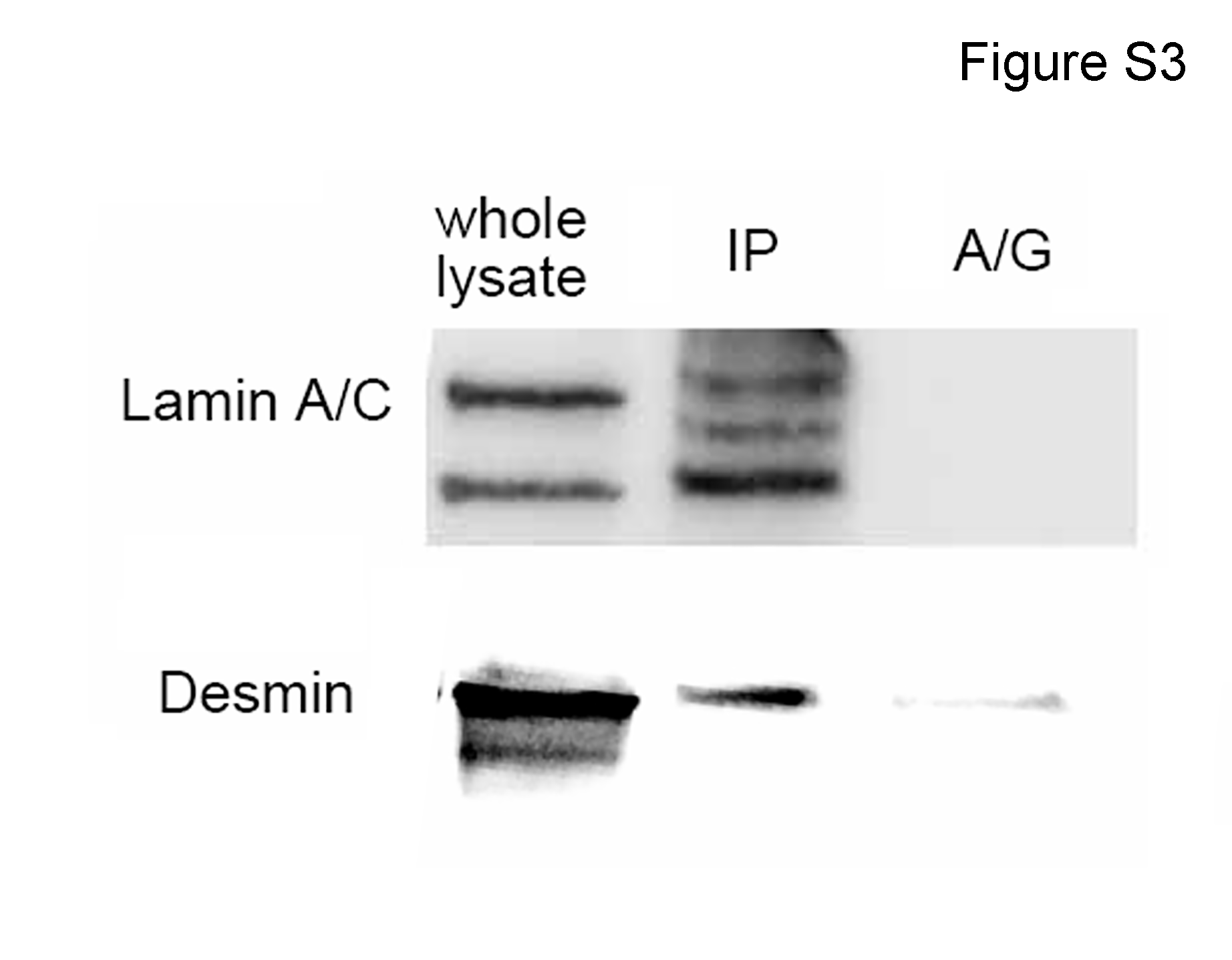

Supplement: Supplementary file 1 [file cells-13-00162-s001.zip › Figure S3.tif]

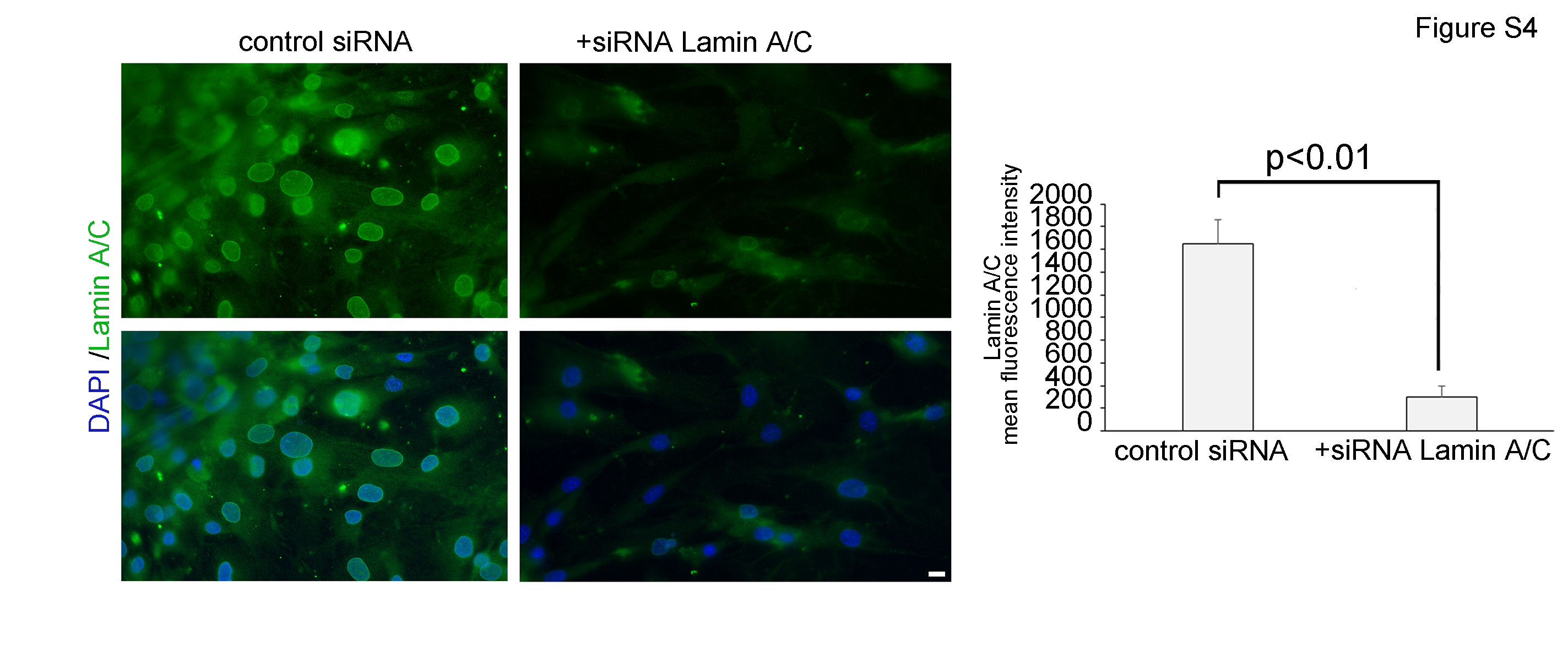

Supplement: Supplementary file 1 [file cells-13-00162-s001.zip › Figure S4.tif]

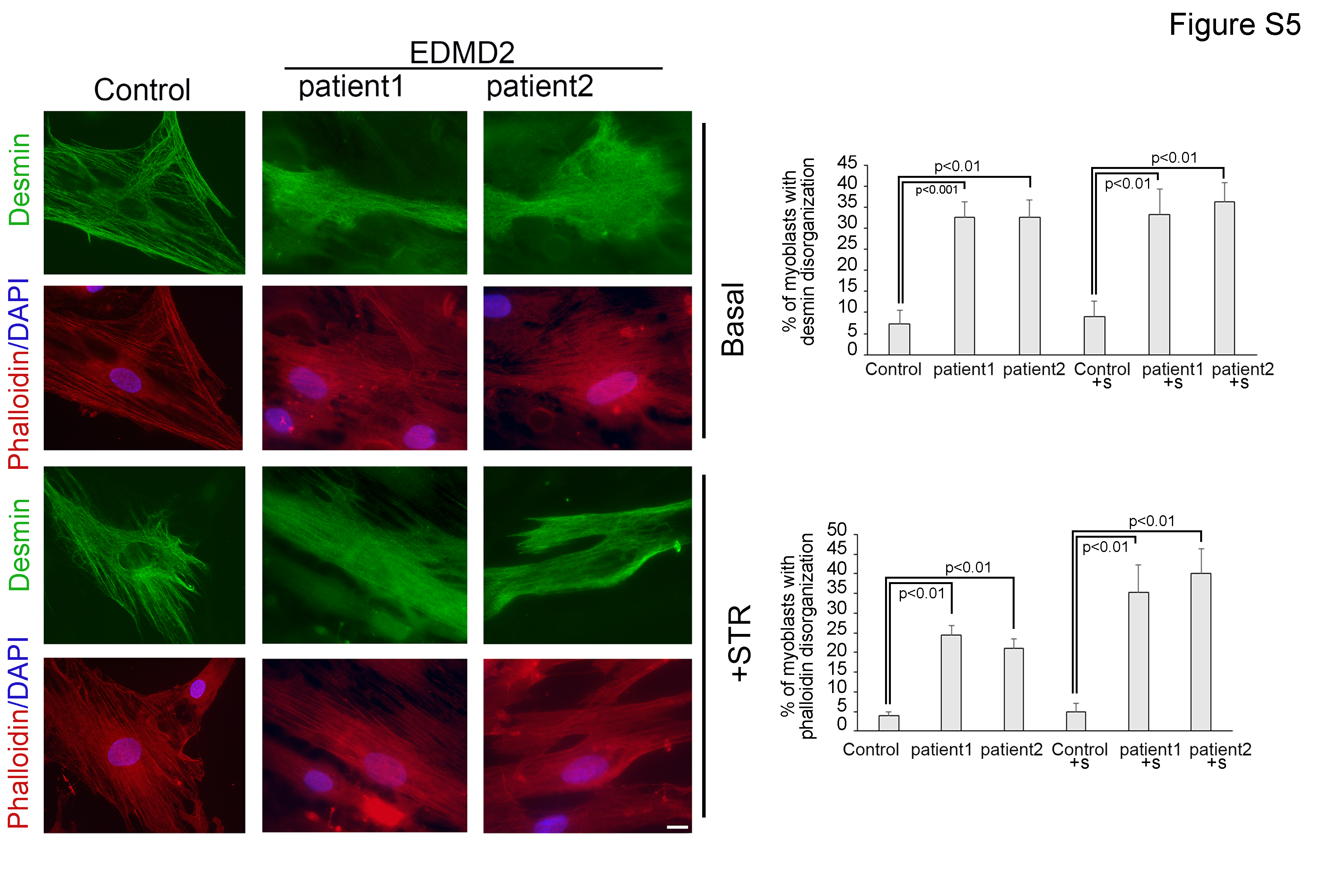

Supplement: Supplementary file 1 [file cells-13-00162-s001.zip › Figure S5.tif]

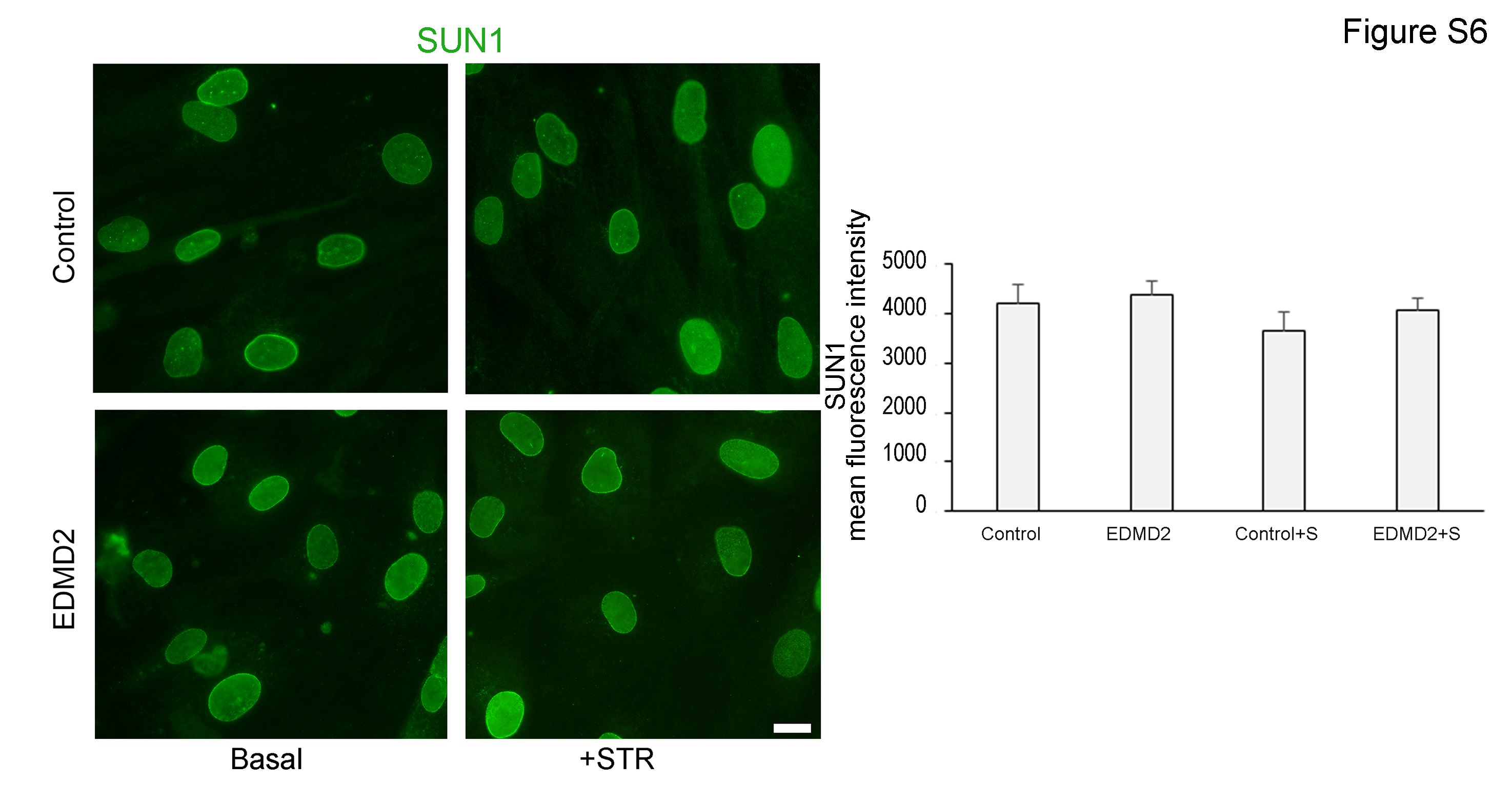

Supplement: Supplementary file 1 [file cells-13-00162-s001.zip › Figure S6.tif]

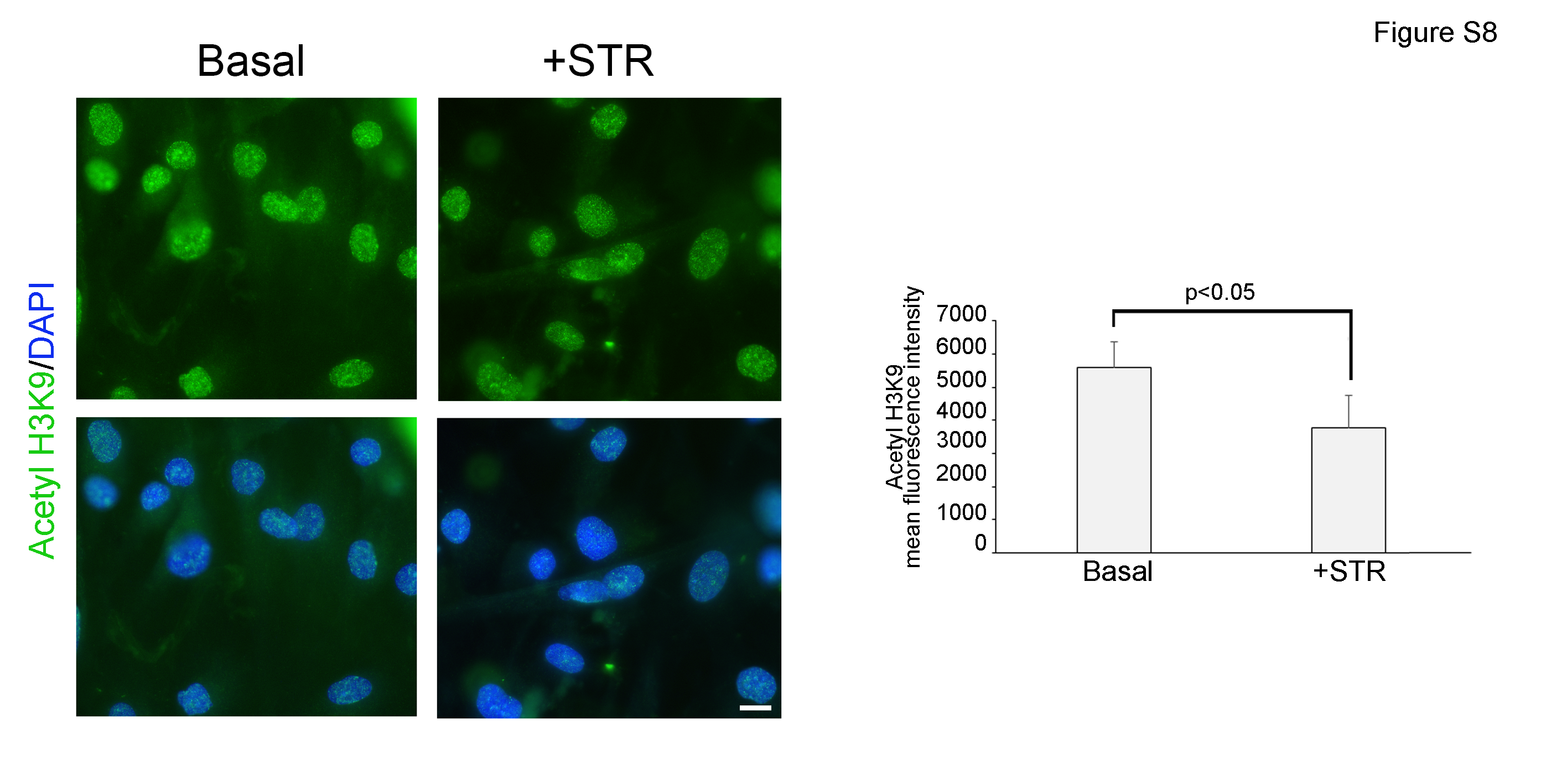

Supplement: Supplementary file 1 [file cells-13-00162-s001.zip › figure S8.tif]
